# Precision Medicine in the treatment of alcohol dependence – the role of gender

**DOI:** 10.1192/j.eurpsy.2025.123

**Published:** 2025-08-26

**Authors:** H. Walter

**Affiliations:** MedUniWien i.R., Vienna, Austria

## Abstract

**Abstract:**

Clinically derived approaches take the different psycho-social and biological conditions into regard and thus render more homogeneous groups, than the current diagnostic criteria, like ICD-11 or DSM 5. Precision medicine, moreover, shows that the amount of drinking, the reason for drinking and thereby also gender seems to be relevant for testing different anticraving drugs. Precision medicine indicates the necessity for more homogeneous subgroups, in order to find differences in the effects of anticraving substances. Many typologies in AUD render more homogeneous subgroups (Lesch et al, 2020). They could increasingly be used for testing anticraving drugs. After presenting these basics in anticraving research, the results of two trials will be presented. First, some genetic results could only be defined in Lesch type 3 female patients (Procopio et al, 2013). Second, in a treatment trial of ondansetron the genetic conditions lead to better sobriety rates only in the not very high drinking group (less than 10 drinks per day) but not in the very high drinking group (Addolorato et al, 2024). Summarizing these research results we see that for anticraving trials we need even more carefully defined subgroups of AUD patients. References: Lesch OM, Walter H, Wetschka Ch, Hesselbrock MN, Hesselbrock V, Pombo S: Alcohol and Tobacco. Medical and Sociological Aspects of Use, Abuse and Addiction. Springer Verlag, 2nd Edition, 2020. Addolorato G, Alho H, Bresciani M, DeAndrade P, Lesch OM, Liu L, Johnson B.: Safety and compliance of long-term low-dose ondansetron in alcohol use disorder treatment. Eur.J.Intern Med, Sept. 127: 43-49; 2024. Procopio DO, Saba LM, Walter H, Lesch O, Skala K, Schlaff G, Vanderlinden L, Clapp P, Hoffman PL, Tabakoff B. Genetic markers of comorbid depression and alcoholism in women. Alc Clin Exp Res. Jun. 37 (6): 896-904, 2013.

**Disclosure of Interest:**

None Declared

